# Time-dependant Bayesian knowledge tracing—Robots that model user skills over time

**DOI:** 10.3389/frobt.2023.1249241

**Published:** 2024-02-26

**Authors:** Nicole Salomons, Brian Scassellati

**Affiliations:** ^1^ Department of Computer Science, Yale University, New Haven, CT, United States; ^2^ I-X and the Department of Computing, Imperial College London, London, United Kingdom

**Keywords:** user modeling, tutoring, human-robot interaction, Bayesian knowledge tracing, robotics

## Abstract

Creating an accurate model of a user’s skills is an essential task for Intelligent Tutoring Systems (ITS) and robotic tutoring systems. This allows the system to provide personalized help based on the user’s knowledge state. Most user skill modeling systems have focused on simpler tasks such as arithmetic or multiple-choice questions, where the user’s model is only updated upon task completion. These tasks have a single correct answer and they generate an unambiguous observation of the user’s answer. This is not the case for more complex tasks such as programming or engineering tasks, where the user completing the task creates a succession of noisy user observations as they work on different parts of the task. We create an algorithm called Time-Dependant Bayesian Knowledge Tracing (TD-BKT) that tracks users’ skills throughout these more complex tasks. We show in simulation that it has a more accurate model of the user’s skills and, therefore, can select better teaching actions than previous algorithms. Lastly, we show that a robot can use TD-BKT to model a user and teach electronic circuit tasks to participants during a user study. Our results show that participants significantly improved their skills when modeled using TD-BKT.

## 1 Introduction

Intelligent Tutoring Systems (ITS) provide one-to-one instruction to a user to increase their knowledge in a particular domain. They can be just as effective as a human tutor VanLehn (2011) under the right circumstances. A robot can enhance an ITS by providing a social presence during the interaction. Compared to a screen system only, embodied robot tutors have been shown to cause greater compliance Bainbridge et al. (2011), higher learning gains Leyzberg et al. (2012), more engagement Wainer et al. (2007), and fewer mistakes Salomons et al. (2022b). A robot can also interact with the user as a peer or tutee rather than as the traditional teacher Salomons et al., 2022a; Chen et al., 2020. Furthermore, a robot has the ability to directly collaborate with the user, and provide demonstrations of the correct answer during more physical tasks (Salomons et al., 2021; Salomons et al., 2022a).

A critical aspect of these systems is to create an accurate model of the user’s skills that estimates which skills the user has mastered and which ones they have not. A skill denotes an ability or a knowledge component in a particular domain. Therefore, different domains will require different skills of the user. When an ITS has an accurate model of a user’s capabilities, it can provide personalized help, focusing on skills that the user has not yet mastered. Systems that provide personalized learning can significantly boost learning in the student VanLehn (2011).

Prior intelligent and robotic tutoring systems have primarily focused on simple tasks such as arithmetic or multiple-choice questions. In these domains, there is a single correct answer. The answer is given either through a tablet or web interface, therefore generating unambiguous observations about the user’s answer. The system uses these end-of-task observations and updates the user skill model depending on whether the answer was correct or incorrect for each skill. For example, if the task tests a division skill by asking: “what is 14/2?” the user will answer 7, a different number, or leave it empty. If the answer was 7, the system increases its estimate about the user’s division skills; otherwise, it decreases it.

Consider a more complex task, such as electronic circuit building or computer programming. These tasks generate opportunities for the system to intervene with help by tracking user skills before the user provides a final answer. Several difficulties arise from modeling throughout task completion. There are often multiple possible ways to complete each task correctly. Frequently these tasks test more than one skill, some of which are expected to be completed earlier than others. Each observation does not tell a complete story about the user’s skills as they apply different skills over time. Lastly, observations can be noisy as sensing systems like computer vision or interpreters are necessary. These are exemplified in a programming task: there are multiple possible solutions; the user needs time to apply each skill, with some skills like creating a loop likely taking longer than others such as creating variables; users will likely break and rebuild pieces of code during the task; the observations are noisy as a language interpreter is necessary.

Previous skill estimation algorithms were not designed to model these more complex tasks. Therefore, this chapter proposes Time-Dependent Bayesian Knowledge Tracing (TD-BKT), which can model a user during more complex domains. There are two main novelties in our proposed solution: an “attempted” parameter that captures the expected amount of time before the user applies each skill. Second, we average the estimates over multiple time-steps. A time-step is the time it takes to get a new observation of the user’s answers. The length of the time-step is determined by the system designer who defines how frequently observations are collected and therefore can vary between different systems or domains. The attempted parameter captures the expected amount of time until the user would have shown their skill if they had mastered it. This means that the system does not immediately assume the user does not know a skill if they do not demonstrate it within the first time-step. The parameter can either be learned through user data, or estimated by an expert in the field. Averaging estimates mean that sensor errors have less of an effect on the estimate. Therefore, the user needs to demonstrate the correct application of a skill multiple times in a row to make decisive conclusions about the user’s skills.

To validate TD-BKT, we compare it against three variations of Bayesian Knowledge Tracing Anderson et al. (1985) (a commonly used method in ITS): the standard BKT model as originally proposed where it is only updated at the end of the task, one where the user model is updated at each time-step based on the model value of the previous time-step, and one where the model is updated from the initial belief at every time-step. We perform three sets of experiments. The first two were done in simulation, where we randomly generated tasks, skills, and users. The first experiment shows that TD-BKT has a more accurate model of the user’s skills throughout the interaction. The second simulation experiment shows that TD-BKT chooses significantly better skills to teach the simulated user than the other algorithms. In our third experiment, we have a robot create a user skill model of participants using TD-BKT and provides tutoring in the domain of electronic circuit skills^1^

## 2 Background

In this section we will provide an overview of the main intelligent tutoring domains in both ITSs and robotics. In sequence we review research in user skill modelling. Lastly we present how ITSs and robots use the user’s skill model to personalize their actions towards the user.

### 2.1 Domains

The domain of an ITS represents the tasks (or problems) that will be given to the user, the skills that compose each task, and each task’s solution. Although some systems can automatically generate tasks and scenarios Niehaus et al. (2011), usually, the domain knowledge is designed by a human expert. The expert designs the skills present in each task and how the system can detect when a skill was demonstrated correctly. Additionally, the domain can include a cognitive model of how to solve each problem and how students proceed with solving it.

Although ITSs have covered a range of domains, they mainly have focused on mathematical domains such as algebra, geometry, and fractions Anderson et al., 1995; Pavlik Jr et al., 2009, or in domains where it is possible to give multiple choice answers Butz et al., 2006; Schodde et al., 2017. These domains are easy to represent in a model as they are composed of factual knowledge. Additionally, there is a single correct answer in these domains, making it straightforward for the user modeling component to model the user’s skills.

Similarly, robotic systems have focused primarily on domains that are easy to represent and model, including geography Jones et al. (2018), nutrition Short et al. (2014), diabetes management Henkemans et al. (2013), and memory skills Szafir and Mutlu (2012). A significant number of studies have also focused on different mathematics subjects including geometry Girotto et al. (2016), arithmetic Janssen et al. (2011), and multiplication Ramachandran et al. (2018). Therefore, there is also a need for us to enable robots to teach a larger variety of domains. Most robots to date are designed with a particular (and usually) singular purpose Vollmer et al. (2016), whereas we need robots that can capture the variety of domains present in the world.

More research should tackle tutoring complex domains, also called ill-defined domains. Fournier-Viger et al. define ill-defined domains as those where traditional tutoring algorithms do not work well Fournier-Viger et al. (2010). They are harder to model because they require more complex representations of skills and correct answers. Domains such as assembling furniture, building an electronic circuit, or creating a computer program can fall under ill-defined domains as modeling algorithms do not capture the user’s skills well in these domains. There are several reasons why these domains can be more challenging to model. Including that they can be order-independent (no clear ordering between skills), they can have multiple solutions, and the tasks are completed over more extended periods.

In recent years, some ITSs and robotic studies have tackled ill-defined domains. Several studies focused on the area of linguistic tutors (Mayo et al., 2000; Weisler et al., 2001), a domain which does not necessarily have one single correct answer and therefore needs a more complex representation. For example, Gordon et al. modeled a child’s reading skills and updated their skill model so a robot could personalize its behaviors Gordon and Breazeal (2015). Some studies focused on teaching programming skills by providing feedback on the user’s code (Abu-Naser 2008; Al-Bastami and Naser 2017). Butz et al. created a tutoring system that taught programming concepts and tested them on multiple-choice questions Butz et al. (2006). Another domain that requires more extended task completion and has order-independent skills is electronic circuits. Graesser et al. studied teaching electronic circuit skills by asking users multiple choice questions in the domain Graesser et al. (2018). Studies used natural language to teach circuits skills Dzikovska et al. (2014) and physics skills Graesser et al. (2001), but these did not estimate the user’s skills.

Despite the growing number of studies done in ill-defined domains, the majority focused on very specific subskills in the domain. Additionally, they frequently limited the user’s responses by asking multiple choice questions or by constraining the environment the user was operating in. Most of these studies did not create a model of the user’s skills during task completion and only observed whether the answer was correct or not. This paper presents an algorithm that can model a user’s skills during an ill-defined domain: electronic circuit building.

### 2.2 User skill modelling

One important aspect of intelligent tutoring systems is assessing which skills the user has mastered and which they have not. With an accurate model of the user’s skills, the system can focus on giving problems and help actions to the user to teach them the skills they have not yet mastered. A system models a user’s skills by observing them respond to various problems. For each problem, it observes whether the user answered correctly. The more problems the student answers correctly, the higher the likelihood that they have mastered that skill. There are several comprehensive reviews of user skill modeling, including (Desmarais and Baker 2012; Pelánek 2017; Liu et al., 2021).

One of the most common methods for determining which skills a user has mastered is Bayesian Knowledge Tracing Corbett and Anderson (1994) (BKT). BKT is a probability-based model in which each skill present in the domain is represented by a probability of mastery. To model the user’s skills, BKT observes whether the student answered correctly and updates the probability of mastery for each skill present in that task. BKT accounts for a student guessing an answer correctly and for a student slipping during a problem (knowing the answer but accidently answering incorrectly) by accounting for probabilities of guessing and slipping. BKT has been extended to account for individual learning differences, including parameterizing each student’s speed of learning to increase the accuracy of the model Yudelson et al. (2013). More details and the equations of BKT are presented in Section 3.1.

An alternative to BKT is Learning Factors Analysis (LFA) Cen et al. (2006), which learns a cognitive model of how users solve problems. It learns each skill’s difficulty and learning rate using user data. However, LFA does not create individualized models for each user and, therefore, cannot track mastery during task completion. Performance Factors Analysis (PFA) Pavlik Jr et al. (2009) addresses LFA’s limitations by both estimating individual user’s skills and creating a more complex model of skills. In recent years, methods based on deep learning have also become prevalent Conati et al. (2002). These generate complex representations of student knowledge. However, this method requires an extensive amount of prior data in the domain Piech et al. (2015).

Although most user skill modeling systems assume a single skill is present in each problem, several models have extended BKT, LFA, and PFA to allow multiple interdependent skills in each problem (Xu and Mostow 2011; González-Brenes et al., 2014; Pardos et al., 2008). However, many multi-skill models assume that all skills must be applied correctly to achieve the correct answer in a problem (Cen et al., 2008; Gong et al., 2010). This is a significant limitation as we do not want the model to assume a user has no mastery over all skills when they might have only failed one. Furthermore, many multi-skill tasks have either order dependencies or knowledge dependencies between skills that need to be accounted for.

BKT, PFA, and deep knowledge tracing are designed to update the model once they have received an unambiguous final answer for the current task. However, this is not the case in more complex tasks where there is noise in the observation, and it takes time for the user to demonstrate each skill in the task. Our proposed solution extends BKT to account for these complexities.

### 2.3 Action selection during tutoring

Once a tutoring systems has an accurate model of a user’s skills, it can use the model to personalize its actions towards the user. The most common way to take advantage of the user model is to determine what task to give a user. For example, Schodde et al. (2017) decides which skill to teach next based on the users’ demonstrated skill. Schadenberg et al., 2017 personalize the difficulty of the content to match the student’s skill (Milliken and Hollinger 2017) chooses the level of autonomy a robot should have depending on the user skills Milliken and Hollinger (2017). Other methods use a modified Partially Observable Markov Decision Process to select which gap (skill) to train the user Folsom-Kovarik et al. (2013), to sequence problems depending on skill difficulty David et al. (2016), and to select tasks that maximizes knowledge of the user’s skill model Salomons et al. (2021).

The system can also personalize its model by providing help to the user during task completion. The system can give many types of help actions, including giving hints, giving an example, a walk-through of the problem, and directly providing the solution to the current problem. Several pieces of work have shown the advantages of choosing personalized help actions (Murray and VanLehn 2006; Rafferty et al., 2016; Clement et al., 2013; Lan and Baraniuk 2016; Yudelson et al., 2013). For example, Ramachandran et al. (2019) decided what type of help to give the user depending on motivation and knowledge.

In these studies, the actions are selected in between tasks or once the user asks for help. However, in complex tasks, there are many opportunities for the system to provide help before the user gives their final answer. Once the system has correctly detected that the user can not complete a skill, it can step in and provide personalized tutoring.

## 3 Time-dependant Bayesian knowledge tracing

In this section we first present the traditional Bayesian Knowledge Tracing (BKT) framework. In sequence, we will review some of the disadvantages that the conventional methods present. Lastly, we present our model called Time-Dependant Bayesian Knowledge Tracing, which solves several of the problems that more complex tasks produce.

### 3.1 Bayesian knowledge tracing

Bayesian Knowledge Tracing (BKT) learns whether a user has mastery of a specific skill by observing the user completing tasks Corbett and Anderson (1994). The estimate of the user’s skill at time *t* is represented by *p* (*L*_*t*_) and is initialized by *p* (*L*_0_) (Eq. 1). Each skill has a probability of being guessed correctly *p*(*G*) and a probability of the user slipping *p*(*S*) (making a mistake despite the skill being known). Additionally, the model has a probability of transitioning (*p*(*T*)) from a non-mastered state to a mastered state whenever the user has an opportunity to try it.

#### 3.1.1 Mastery probability initialization

The probability of mastery of the user is set to its prior at the start of the interaction (Eq. 1).

$$p\left(L_{1}\right)=p\left(L_{0}\right)$$ (1)

#### 3.1.2 Mastery probability update

The model observes whether the user got the correct or incorrect answer after completing the task and uses it to update the probability of mastery. To update the mastery when the observation is incorrect (Eq. 4), the new estimate is the prior times the probability that they slipped, divided by the total probability of an incorrect answer (Eq. 2). When the observation is correct (Eq. 5), the updated probability of mastery is the prior probability of mastery times the probability that they did not slip, divided by the total probability of a correct answer (Eq. 3).

$$p\left(o_{t}=0\right)=p\left(L_{t}\right)\cdot p\left(S\right)+\left(1-p\left(L_{t}\right)\right)\cdot \left(1-p\left(G\right)\right)$$ (2)

$$p\left(o_{t}=1\right)=p\left(L_{t}\right)\cdot \left(1-p\left(S\right)\right)+\left(1-p\left(L_{t}\right)\right)\cdot p\left(G\right)$$ (3)

$$p\left(L_{t}|o_{t}=0\right)=\frac{p\left(L_{t-1}\right)\cdot p\left(S\right)}{p\left(o_{t}=0\right)}$$ (4)

$$p\left(L_{t}|o_{t}=1\right)=\frac{p\left(L_{t-1}\right)\cdot \left(1-p\left(S\right)\right)}{p\left(o_{t}=1\right)}$$ (5)

#### 3.1.3 Transition probability

The probability of the user going from an non-mastered state to a mastered state is calculated from the probability of them already having mastered the skill plus the probability of them not having mastered the skill times the probability of them transitioning (Eq. 6).

$$p\left(L_{t+1}\right)=p\left(L_{t}|o_{t}\right)+\left(1-p\left(L_{t}|o_{t}\right)\right)\cdot p\left(T\right)$$ (6)

### 3.2 Bayesian knowledge tracing limitations

The BKT model was designed for tasks where unambiguous observations of the user are given at the end of each task. It would be advantageous for the system to create an accurate model and provide help throughout the task. With some simple modifications, the BKT expression could be adapted to allow for continuous modeling. One option would be to use the BKT update equations after every time-step. However, this quickly brings the estimate to one of the extremes (*p* (*L*_*t*_) = 0 or *p* (*L*_*t*_) = 1), especially if many same observations are seen in a row. Another option is to update it every time-step using the initial mastery estimate (*L*_0_). When doing this, the mastery jumps between high and low mastery every time the observations change. Furthermore, neither of these two proposed solutions considers whether the user is currently at the start or end of the task. Towards the end of the task, the user has had more time to demonstrate their skill mastery.

### 3.3 TD-BKT

We propose an extension of BKT that continuously updates its estimate of the user’s skills during task completion. We call it Time-Dependant Bayesian knowledge Tracing (TD-BKT). In addition to the BKT parameters, we introduce a new variable called *attempted*. The attempted parameter (*E* [*k*]) is the expected number of time-steps it would take for the user to have had time to attempt the skill *k*. It can be estimated from prior data or by an expert in the field. For example, in the programming domain, we would not expect the user to have completed a FOR loop after the first second of the task. Rather, it would likely take several minutes to attempt it. On the other hand, creating a new variable would likely be attempted in the first minute. Therefore, the attempted parameter accounts for these differences in time needed, and updates the model relatively for each parameter.

An additional modification to BKT is that we average the *n* previous time-steps to determine the current estimate of the user’s skills. There are two main advantages to averaging the skills. The first is that “breaking” part of the task during completion has a smaller effect on the model. For example, when programming, you might need to move code into a new function, temporarily creating non-functioning code. The second advantage is that noise has a much smaller effect on the model. When using computer vision systems or natural language processing, it is common to have occasional observation errors. But these errors are mostly nullified when averaging with the correct observations.

#### 3.3.1 Probability of an observation

We separate the observations into two cases: when the user has already attempted the skill, and when they have not. When the user has attempted the current task, the probabilities of the correct and incorrect observation are identical to BKT (Eqs 7, 8). When they have not attempted it, the probability of an incorrect observation is guaranteed, whereas the probability of a correct observation is zero (Eqs 9, 10).

$$p\left(o_{t}=0|A=1\right)=p\left(L\right)\cdot p\left(S\right)+\left(1-p\left(L\right)\right)\cdot \left(1-p\left(G\right)\right)$$ (7)

$$p\left(o_{t}=1|A=1\right)=p\left(L\right)\cdot \left(1-p\left(S\right)\right)+\left(1-p\left(L\right)\right)\cdot p\left(G\right)$$ (8)

$$p\left(o_{t}=0|A=0\right)=1$$ (9)

$$p\left(o_{t}=1|A=0\right)=0$$ (10)

#### 3.3.2 Attempted probability

The probability of a skill *k* having been attempted is the current time-step divided by the number of expected time-steps to complete it. If the number of time-steps passed has exceeded the attempted parameter, it is assumed that the user would have attempted it if they had mastered that skill (Eq. 11).

$$P\left(A|t\right)=\left\{\begin{array}{c} \begin{array}{cc} \frac{t}{E\left[k\right]}, & \text{if }t\leq E\left[k\right]\\ 1, & \text{if }t>E\left[k\right] \end{array}\; \end{array}\right.$$ (11)

#### 3.3.3 Mastery probability initialization

Similar to BKT, the probability of mastery is equal to the prior estimate (Eq. 12). However, contrary to BKT, it will not change over time. *p*(*L*) will be used to update the current temporary mastery over time *P*(*H*_*t*_).

$$p\left(L\right)=p\left(L_{0}\right)$$ (12)

#### 3.3.4 Mastery probability update

As seen in Eq. 13, instead of looking at each time-step individually, the algorithm updates its current estimate (*p* (*H*_*t*_)) by averaging the previous *n* time-steps. At each time-step. if the observation is that the user applied the skill correctly, then the task must have been attempted, and the traditional BKT equation is used (Eq. 14). When the observation is incorrect, there are two possibilities: either the task has been attempted, but the person did not demonstrate the skill, or the task has not been attempted yet. Eq. 15 measures the probability of mastery considering both scenarios and divides it by the total probability of an incorrect observation. Here we denote *t* as the current time-step, and *i* as the variable iterating through the previous *n* time-steps.

$$p\left(H_{t}\right)=\overset{t}{\underset{i=t-n}{\sum }}p\left(H_{i}|L,o_{i},i\right)$$ (13)

$$p\left(H_{i}|L,o_{i}=1,i\right)=\frac{p\left(L\right)\cdot \left(1-p\left(S\right)\right)}{p\left(o_{i}=1|A=1\right)}$$ (14)

$$p\left(H_{i}|L,o_{i}=0,i\right)=\frac{p\left(L\right)\cdot \left[p\left(A|i\right)\cdot p\left(S\right)+\left(1-p\left(A|i\right)\right)\right]}{p\left(A|i\right)\cdot p\left(o_{i}=0|A=1\right)+\left(1-p\left(A|i\right)\right)}$$ (15)

#### 3.3.5 Derivations

Below we provide the derivations of how the mastery probability is updated given the observation at time-step *i* and considering the probability that the task has been attempted. First, we consider the case when we see that the user demonstrates the skill (*o*_*i*_ = 1).

$$p\left(H|L,o_{i}=1\right)=\frac{p\left(L\right)p\left(o_{i}=1|L\right)}{p\left(o_{i}=1\right)}$$

$$\begin{array}{ll} & p\left(H|L,o_{i}=1\right)\\ & =\frac{p\left(L\right)*\left[p\left(o_{i}=1|L,A=1\right)p\left(A=1\right)+p\left(o_{i}=1|L,A=0\right)p\left(A=0\right)\right]}{p\left(o_{i}=1|A=1\right)p\left(A=1\right)+p\left(o_{i}=1|A=0\right)p\left(A=0\right)} \end{array}$$

$$p\left(H|L,o_{i}=1\right)=\frac{\left.p\left(L\right)*\left(1-p\left(S\right)\right)p\left(A=1\right)+0*p\left(A=0\right)\right]}{p\left(o_{i}=1|A=1\right)p\left(A=1\right)+0*p\left(A=0\right)}$$

$$p\left(H|L,o_{i}=1\right)=\frac{p\left(L\right)*\left(1-p\left(S\right)\right)p\left(A=1\right)}{p\left(o_{i}=1|A=1\right)p\left(A=1\right)}$$

$$p\left(H|L,o_{i}=1\right)=\frac{p\left(L\right)\left(1-p\left(S\right)\right)}{p\left(o_{i}=1|A=1\right)}$$

Here we consider the case when we see the user did not demonstrate the skill (*o* = 0).

$$p\left(H|L,o_{i}=0\right)=\frac{p\left(L\right)p\left(o_{i}=0|L\right)}{p\left(o_{i}=0\right)}$$

$$\begin{array}{ll} & p\left(H|L,o_{i}=0\right)\\ & =\frac{p\left(L\right)*\left[p\left(o_{i}=0|L,A=1\right)p\left(A=1\right)+p\left(o_{i}=0|L,A=0\right)p\left(A=0\right)\right]}{p\left(o_{i}=0|A=1\right)p\left(A=1\right)+p\left(o_{i}=0|A=0\right)p\left(A=0\right)} \end{array}$$

$$p\left(H|L,o_{i}=0\right)=\frac{p\left(L\right)*\left[p\left(S\right)p\left(A=1\right)+1*p\left(A=0\right)\right]}{p\left(o_{i}=0|A=1\right)p\left(A=1\right)+1*p\left(A=0\right)}$$

$$p\left(H|L,o_{i}=0\right)=\frac{p\left(L\right)\left[p\left(A\right)p\left(S\right)+\left(1-p\left(A\right)\right)\right]}{p\left(A\right)p\left(o_{i}=0|A=1\right)-\left(1-p\left(A\right)\right)}$$

## 4 Comparison to traditional methods

In this section we will provide an intuitive toy example of how different algorithms update the model of a user’s skill. We compare TD-BKT to several variations of traditional Bayesian Knowledge Tracing models given a specific observation. We will compare the following models:

• Traditional BKT (T-BKT)—The user’s skill estimate is only updated at the end of the task, using the final observation.
• Initial BKT (I-BKT)—Modification of the BKT model, where it updates its current estimate using the initial belief value during each time-step.
• Every time-step BKT (E-BKT)—Modification of the BKT model, where it uses the user’s skill estimate from the previous time-step to update the value of the current time-step.
• Time-Dependant BKT Only Attempted (TD-BKT-AT)—The TD-BKT model with only the attempted parameter (presented in Eq. 11. This does not include TD-BKT’s averaging of beliefs.
• Time-Dependant BKT Only Average (TD-BKT-AV)—The TD-BKT model, but only averaging the beliefs over time (Eqs 13–15). In this case it averages the previous 10 time-steps. This model does not include TD-BKT’s attempted parameter.
• TD-BKT—Our proposed algorithm using both the attempted and the averaging parameters.

First, we give an intuitive demonstration via a toy example of the pitfalls of traditional BKT when the interaction is multiple time-steps long. We graphically show how TD-BKT mitigates some of those problems. Let us consider a task where a person is building an electronic circuit that requires a resistor and the user is given 60 time-steps to complete the task. The user adds the resistor at time-step 22, removes it at time-step 30, and then returns it to the same position at time-step 45 for the remainder of the time. The observation is 0 (incorrect) when the resistor is not on the board and is 1 (correct) when the resistor is on it. We set that the expectation of how long it will take to add the resistor to the circuit is 60 time-steps (*E* [*k*] = 60). We set the prior probability of the user having mastered this skill to complete uncertainty (*P* (*L*_0_) = 0.5). Lastly, we set the probability of guessing and the probability of slipping to 0.1 (*P*(*G*) = *P*(*S*) = 0.1).

**FIGURE 1:**
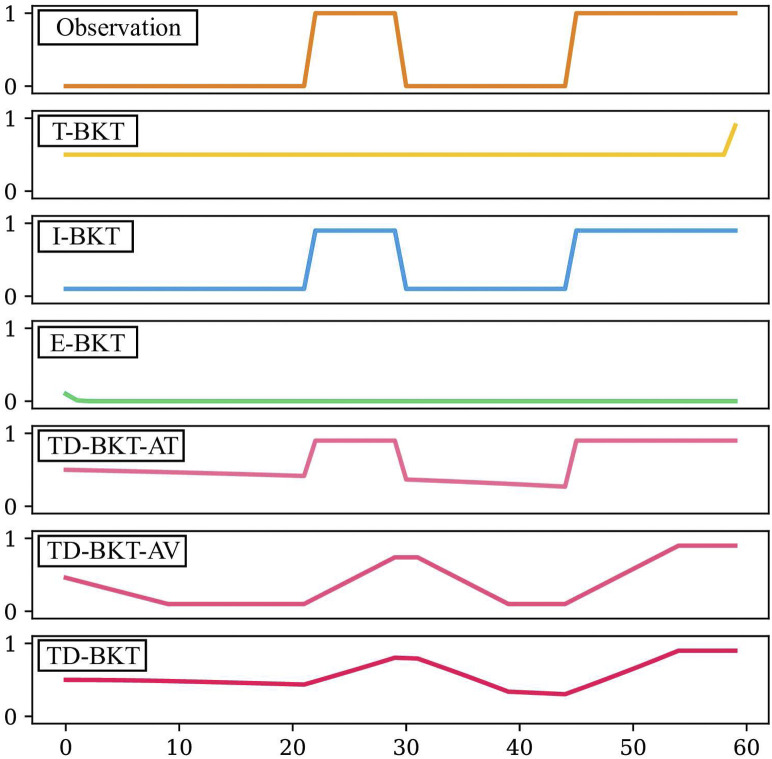
A comparison of how different variations of traditional BKT update the belief of a particular skill after specific observations at each time-step. We also demonstrate the effect of the different elements of TD-BKT and how the final TD-BKT updates its belief.

In Figure 1, the TD-BKT and conventional BKT methods are compared with respect to their belief of the resistor skill over the task completion. T-BKT is shown to update only at the end, which means it loses the opportunity to make informed decisions throughout the task. I-BKT jumps between higher and lower belief states with correct or incorrect observations, since it uses the initial belief to update rather than using any history. Lastly, because E-BKT is updated every time-step, when several incorrect observations are made in a row at the start, it quickly brings the belief to zero. It would need many correct observations to recover.

We can observe the effect of the attempted parameter in the trace for the TD-BKT-AT approach. The belief lowers very slowly at the start (as the person has likely not had an opportunity to demonstrate their skills yet) and then decreases faster when more time-steps have passed. The TD-BKT-AV approach shows the result of averaging the current belief of the previous ten time-steps. Instead of jumping from high to low states, it takes several rounds of the same observation to impact the belief significantly. Finally, TD-BKT shows the result of the attempted parameter and the average combined. The model creates a smoother model of the user’s skills and considers how far along the user is in the task.

## 5 Simulation

We examine the presented algorithms under two experimental conditions. The first focused on user modeling and the second on the effects of using the skill model to choose teaching actions. The performance of each algorithm is examined across 1000 rounds of simulated tasks, each initialized with randomized skills, tasks and users.

Skills—During each round, different skills were created. Each skill had associated with it a probability of guessing and a probability of slipping, randomly chosen from a uniform distribution between 0.1 and 0.25. The amount of time the user needed to expect to complete a skill was set to a random uniform distribution between 40 and 150.

Tasks—During each round, a new task was created. The task was assigned between five and ten skills. Each task was given 180 time-steps for completion.

User—During each round, a simulated user was generated. For each skill, they were randomly assigned as mastering that skill or not with equal probability. We specify as *T* ^*i*^ the true state of the user for skill *i*. The belief state *b* of the user was set to 0.5 (the model had complete uncertainty) for all skills at the start of the round.

Observations—During each time-step an observation is generated for the user. The observation was generated via the probability of a correct or incorrect observation (Eqs 7–10) given their mastery in the skill, times the probability of the skill having been attempted (Eq. 15).

Teaching—Every 20 time-steps, the user is taught one of the skills. The chosen skill is the one with the lowest estimated mastery state. The probability of learning a skill (when it was not previously known) is randomly drawn from a uniform distribution between 0.15–0.35. If they have learned it, then their mastery of that skill goes from 0 to 1.

### 5.1 Experiment 1: user skill modeling

In the first experiment, we compare the accuracy of TD-BKT’s skill model with the estimates of T-BKT, I-BKT, and E-BKT. In Experiment 1, we assume that no teaching has occurred and focused on skill modeling accuracy. To measure how well each algorithm performs, we calculate for each skill *i* how far the estimate from the model (represented by belief *b*) is from the user’s true skill state *T* ^*i*^ is at every time-step. We measure the distance between the belief at time-step *t* and the true state of the user (Eq. 16) using Kullback-Leibler Divergence (KLD) Kullback and Leibler (1951). KLD is used as it measures how different two probability distributions are from each other. Therefore the smaller the KLD, the more similar the belief is to the true skill state.

$$D\left(b,T\right)=\underset{i\in skills}{\sum }b_{t}^{i}\cdot log\frac{b_{t}^{i}}{T^{i}}+\left(1-b_{t}^{i}\right)\cdot log\frac{1-b_{t}^{i}}{1-T^{i}}$$ (16)

**FIGURE 2:**
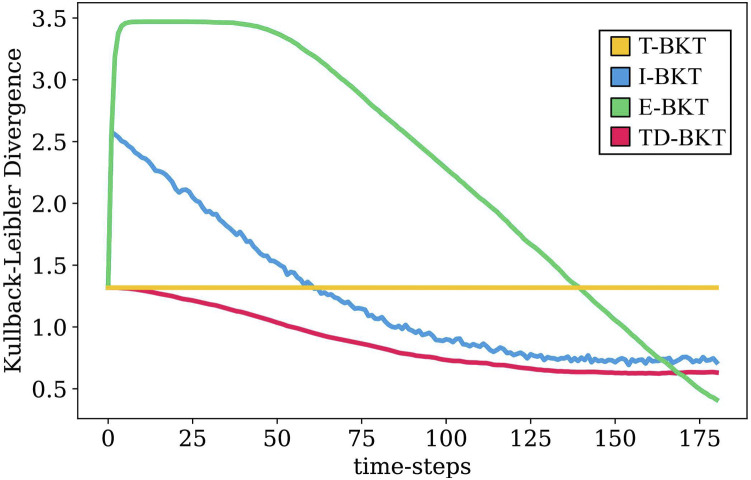
In this graph we present the average Kullback-Leibler Divergence distances of the 1000 rounds of simulation. We rpesent the KLD of the user’s estimated skill state and their real skill state for our proposed model and three variations of Bayesian Knowledge Tracing. As seen in the graph, TD-BKT outperforms the other algorithms and more quickly created an accurate model of the user’s skills.

Figure 2 shows the KLD of estimate *b* at each time-step for the different BKT variations. T-BKT only updates its belief at the end, and therefore remains constant throughout the interaction. At the start, E-BKT performs the worst of all the algorithms but corrects its mistakes at the end when observations are more reliable. Both TD-BKT and I-BKT improve their estimates as time progresses. However, I-BKT initially diverges from the true skill state by giving a large amount of weight to initial observations despite them being very unreliable as the user has not had time to demonstrate any of the skills yet. On the other hand TD-BKT gives less weight to initial observations and outperforms the other models during most time-steps.

At four different time-steps (time-step 30, 80, 130, and 180), we measured the KLD skill accuracy. The means and standard deviations for each of the algorithms are presented in Table 1. We measured whether the KLD skill accuracy was significantly different between the different models using an ANOVA with a *post hoc* Tukey HSD test. At all different time points, the different models were statistically significant from each other (time-step 30: *F* (3, 3996) = 1.03, *p* < 0.001; time-step 80: *F* (3, 3996) = 0.94, *p* < 0.001; time-step 130: *F* (3, 3996) = 0.53, *p* < 0.001; time-step 180: *F* (3, 3996) = 0.63,*p* < 0.001). Table 2 shows the *p*-values for each pairwise comparison and the effect-sizes (Cohen’s d values) for each pairwise comparison. TD-BKT significantly outperforms T-BKT, I-BKT, and E-BKT after 30, 80, and 130 time-steps. However, after 180 time steps, I-BKT had a better model of the user’s skills.

**TABLE 1:** Means and standard deviations for KLD for each of the algorithms at time-steps 30, 80, 130, and 180.

	ts 30	ts 80	ts 130	ts 180
**T-BKT**	M: 1.33	M: 1.33	M: 1.33	M: 1.33
	SD: 0.55	SD: 0.55	SD: 0.55	SD: 0.55
**I-BKT**	M: 1.94	M: 1.06	M: 0.77	M: 0.73
	SD: 0.95	SD: 0.73	SD: 0.58	SD: 0.55
**E-BKT**	M: 3.50	M: 2.79	M: 1.55	M: 0.44
	SD: 1.31	SD: 1.27	SD: 1.11	SD: 0.62
**TD-BKT**	M: 1.20	M: 0.85	M: 0.66	M: 0.63
	SD: 0.52	SD: 0.42	SD: 0.34	SD: 0.33

**TABLE 2:** The table includes the *p*-values for each pairwise comparison using an ANOVA with Tukey HSD Corrections, at time-steps 30, 80, 130, and 180. It also includes the pairwise effect sizes for each comparison.

ts 30	I-BKT	E-BKT	TD-BKT	ts 80	I-BKT	E-BKT	TD-BKT
**T-BKT**	*p* < 0.001	*p* < 0.001	*p* = 0.004	**T-BKT**	*p* < 0.001	*p* < 0.001	*p* < 0.001
	d = 0.79	d = 2.16	d = 0.24		d = 0.42	d = 1.49	d = 0.98
**I-BKT**		*p* < 0.001	*p* < 0.001	**I-BKT**		*p* < 0.001	*p* < 0.001
		d = 1.36	d = 0.97			d = 1.67	d = 0.35
**E-BKT**			*p* < 0.001	**E-BKT**			*p* < 0.001
			d = 2.31				d = 2.05

**ts 130**	**I-BKT**	**E-BKT**	**TD-BKT**	**ts 180**	**I-BKT**	**E-BKT**	**TD-BKT**
**T-BKT**	*p* < 0.001	*p* < 0.001	*p* < 0.001	**T-BKT**	*p* < 0.001	*p* < 0.001	*p* < 0.001
	d = 0.42	d = 1.49	d = 0.98		d = 1.09	d = 1.52	d = 1.54
**I-BKT**		*p* < 0.001	*p* = 0.005	**I-BKT**		*p* < 0.001	*p* < 0.001
		d = 1.67	d = 0.23			d = 0.50	d = 0.22
**E-BKT**			*p* < 0.001	**E-BKT**			*p* < 0.001
			d = 1.08				d = 0.38

### 5.2 Experiment 2: skill modeling with teaching

During Experiment 2, the simulated user was taught a skill every 20 time-steps. For each model, the chosen skill to teach was always the one with the lowest estimated belief. To measure how much a simulated user has learned, we measure the number of skills they had mastered at the start of the interaction (time-step 0) compared to the number of skills they had mastered at the end of the round (time-step 180) using Eq. 17. We use the true skill state *T* for the calculation. We also measure how many skills the user would have learned if the system had a perfect model at each time-step of the user’s skills. We call this the optimal model, as it can choose the best skills to teach.

$$D\left(T_{\mathrm{start}},T_{\mathrm{end}}\right)=\underset{s\in skills}{\sum }T_{s}^{\mathrm{end}}-T_{s}^{\mathrm{start}}$$ (17)

**FIGURE 3:**
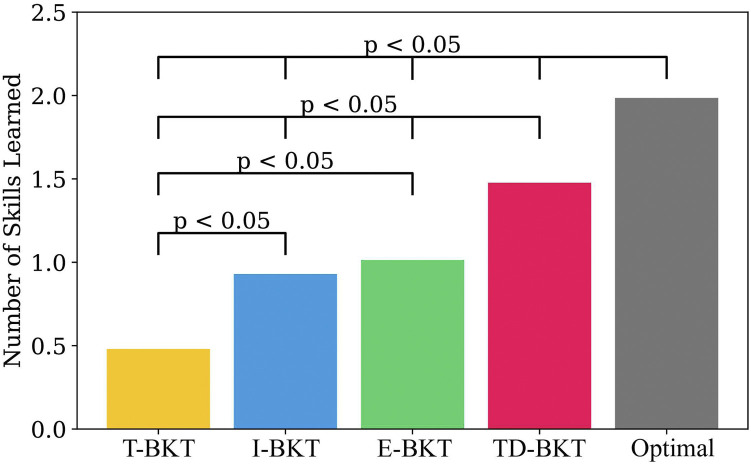
In this graph we present TD-BKT and the BKT variations with respect to the average number of skills learned over 1000 time-steps. Simulated users improved their skills significantly more using TD-BKT’s skill model than when the other BKT variations were used. However the Optimal model (where perfect knowledge of the user’s skills is known beforehand) performs the best.

Figure 3 shows the number of skills the user has learned on average for TD-BKT and the different BKT variations. On average, the simulated user’s in T-BKT learned 0.42 (*SD* = 0.49) new skills; users in I-BKT learned 0.91 (*SD* = 0.57) new skills; users in E-BKT learned 1.00 (*SD* = 1.15) new skills; users in TD-BKT learned 1.44 (*SD* = 0.82) new skills; and users with the Optimal model learned 1.89 (*SD* = 1.15) new skills. The models differed statistically significantly using an ANOVA with *post hoc* Tukey HSD Test *F* (4, 4995) = 0.65, *p* < 0.001. All two-pair comparisons were statistically significant (*p* < 0.001), other than between the I-BKT and the E-BKT models (*p* = 0.155). All *p*-values and effect sizes (Cohen’s d) are shown in Table 3. TD-BKT outperforms all the BKT variations, and is only behind the optimal model.

**TABLE 3:** The table includes the *p*-values for each pairwise comparison using an ANOVA with Tukey HSD Corrections, for the number of skills learned. It also includes the pairwise effect sizes for each comparison.

Skills learned	I-BKT	E-BKT	TD-BKT	Optimal
**T-BKT**	*p* < 0.001	*p* < 0.001	*p* < 0.001	*p* < 0.001
	d = 0.92	d = 1.15	d = 1.44	d = 1.64
**I-BKT**		*p* = 0.155	*p* < 0.001	*p* < 0.001
		d = 0.14	d = 0.72	d = 1.04
**E-BKT**			*p* < 0.001	*p* < 0.001
			d = 0.65	d = 0.98
**TD-BKT**				*p* < 0.001
				d = 0.43

## 6 User study

In this section we have a user study where a robot uses TD-BKT on a real task with human participants. The main goals of this section are to demonstrate how to apply TD-BKT to a real task, by designing appropriate skills and tasks. We demonstrate how TD-BKT can properly model a variety of different users through a task where observations are noisy. We also show that TD-BKT selects relevant help actions and show that participants increase their skills throughout the session. More details on the user study can be found in Salomons et al. (2022a).

**FIGURE 4:**
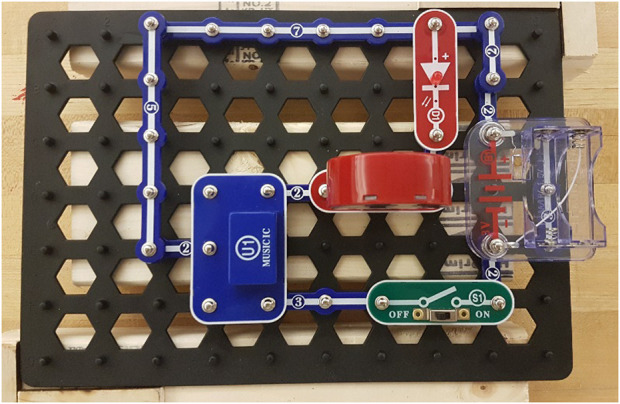
An example of a completed circuit. This circuit plays music and blinks a light in the rhythm of the music, when the switch is turned on.

The chosen task for our user study was electronic circuit. We use snap circuits Elenco (2021), where the pieces can be snapped together on a board to form circuits. An example of a built snap circuit board can be seen in Figure 4. Electronic circuits encapsulate well how to model skills during more complex tasks, as there are multiple correct ways to create a circuit. Users will be adding, moving, and removing pieces on the board during the interaction. Moreover, the observations are noisy since a computer vision system detects what pieces are added to a circuit board. Lastly, it often takes many minutes for a participant to complete a singular circuit.

Skills—There were eight different pieces that a person could add to a board: a switch, a button, a resistor, an LED, a music circuit, a speaker, a motor, and wires. Knowing when to add each of these different pieces to the board was considered a skill. Additional skills included knowing how to create a closed loop circuit, knowing the directionality of an LED, how to create AND and OR gates, how to connect the different ports of a music circuit piece, and so on. We tested a total of 17 different skills. The parameters for each skill (slipping and guessing probability, and the attempted parameter) were determined by consulting an electronic engineering major.

Tasks—We created different tasks to test different combinations of snap circuit skills. Participants were given an empty board with only a battery on it and given 3 minutes for each task unless they correctly completed it before the time. Some examples of tasks were: “Build a circuit that plays music when a switch is turned on” and “Build a circuit that spins a motor when a switch is turned on or a button is pressed”. Each task had a degree of difficulty associated with it, and the next task was chosen according to the user’s skill. There were 32 variations of tasks, of which each user completed 10. Our algorithm chose the next task to present the user depending on the model that TD-BKT has built. The tasks were chosen so that they were not too difficult and not too easy. Participants were told which task to complete next via an application on a tablet. More details on how the robot chose which task to give is presented in section ??

Users—There were 37 participants in the experiment (18 male, 18 female, 1 non-binary). The study was approved by the university’s Institutional Review Board, and participants signed a consent form. They were not provided with any information on how electronic circuits worked, other than the piece’s name and the ports on the pieces. Participants completed a pre-test and a post-test to determine their knowledge of circuits before and after the interaction.

Observations—An overhead camera observed the user as they completed each task. A vector of observations was generated at each time-step for the task. If the user demonstrated the correct skill, the observation for that skill would be 1; if they did not demonstrate the skill, it would be 0; and if a skill was not tested during that task, it would be a 2.

Teaching—Every 30 s, a robot provided help. The help action varied between pointing out wrong pieces on the board, suggesting pieces to add, explaining how to connect pieces, and affirming that a skill they had demonstrated was correct. The user had the option to press a “finished” button on a tablet. Upon indicating they had finished, the robot would provide further help if one of the skills was incorrect. If the task was correct, it would move on to the next task. More details on how the robot chose which skill to teach is presented in Section 6.2.

### 6.1 Robot system

**FIGURE 5:**
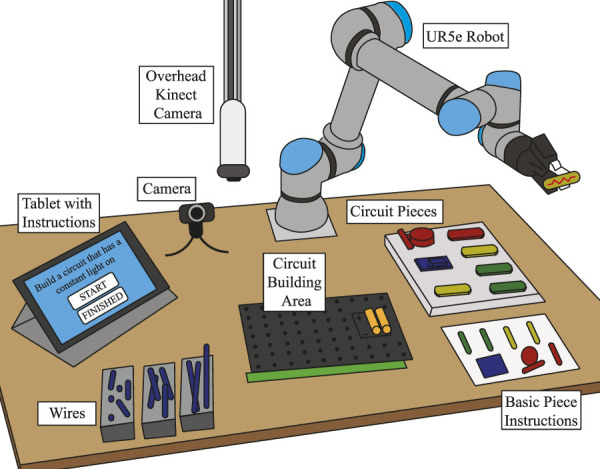
The experimental setup. Participants were given tasks via a tablet application. In the middle of the table, they built circuits using wires and circuit pieces. They were provided basic instructions with the piece names. An overhead camera focused on the circuit and modeled which skills were correctly applied. A UR5e robot provided them with help every 30 s based on what was needed for the current task.

Participants interacted with the robot on a large table. Figure 5 shows an illustration of the experimental setup. Participants were given each task via a tablet, and on the tablet, they could indicate that they had finished the current task and start the next task. The tablet provided no help with the task. Participants used wires and electronic circuit pieces to build their circuits on a board in the middle of the table. An overhead Kinect Azure camera detected what pieces were on the board and how they were connected. A green hand strip at the bottom of the board was used to detect when the participants’ hands were on top of the board, and therefore the camera’s observations would be inaccurate.

A UR5e robot from Universal Robots was used in this study. It is a lightweight industrial robotic arm with 6-DOF. It could pick up the snap circuit pieces with its gripper and hand them to the participant. The robot was able to communicate to the participant via a text-to-speech voice. Additionally, the robot displayed idling behavior with random movements every few seconds, occasionally looking at the circuit board, pieces, or the participant, by pointing the gripper at it. The robot acted completely autonomously throughout the study.

### 6.2 Task selection

Prior work shows that selecting tasks with appropriate difficulty leads to higher learning gains David et al., 2016; Salomons et al., 2021. Therefore tasks were chosen for each participant according to their demonstrated capabilities. To rate the difficulty of each task, each of the 17 skills was given a difficulty rating from a scale of 1.0–5.0, with 5.0 being the most difficult. These were determined by consulting an electronic engineering major. The ratings were stored in a difficulty vector *d*. For example, the skill for whether a participant knew when to use an LED was given a difficulty rating of 1, while the skill for whether the participant knew how to create an OR gate was given a 4.5. The current belief estimate *b* was used to select the next task.

In order to determine which task to give next to a participant, all remaining tasks are assigned a difficulty rating *R* based on the skills *Sk* that a task *t* incorporated. The rating was calculated based on the difficulty of each skill and the participant’s current belief value *b*. Participants with higher belief values would likely find the task easier. Therefore, we used 1 − *b*(*i*) to measure how difficult the task would be for the participant. As we are summing over the difficulty of each skill for a task, the more skills a task tests, the more difficult it will likely be. The difficulty rating *R* for a specific task is calculated as follows:

$$R_{t}=\underset{i\in Sk}{\sum }\left(1-b\left(i\right)\right)\;*\;d\left(i\right)$$ (18)

There is also a fixed ideal rating value *V* that was set equal to five after initial trial and error. The *V* is intended to help ensure that an appropriate task is selected next for the respective participant so that the task is not too easy nor too overwhelming Metcalfe and Kornell (2005). The task whose *r* value is closest to *V* is selected as the next task and removed from the possible remaining tasks for the next iteration.

$$NextTask=\underset{t\in T}{\min}\left(\left|R_{t}-V\right|\right)$$ (19)

In the case where several tasks are equally close to *V*, one of these potential tasks is selected at random. The process is repeated until the interaction with the participant ends.

### 6.3 Finished signal

One simple addition to TD-BKT was that the user could signal via a tablet when they were finished with the task. We interpret the user pressing the button, as signalling that they have attempted all the skills. Therefore, when the participant pressed the button, we update the prior p(L) with Eq. 20.

$$p\left(L\right)=P\left(H_{t}|o_{t}\right)$$ (20)

### 6.4 Pre-test and post-test

The pre-test and post-test were composed of six very similar questions. The first two questions on both tests were the same. They asked participants to build from scratch a circuit that shines a constant light and a circuit that plays music, respectively. Participants were given 5 minutes to do both tasks. The third and fourth tasks on both tests required participants to add pieces to the board to complete the circuits. These tasks were identical between pre-test and post-test, other than the circuit boards being rotated 180° to the participant in the post-test. For the fifth and sixth tasks, we presented pictures of pre-built circuits and asked participants to write down what the circuits did. These were similar between pre-test and post-test, but the pieces were arranged differently on the board. Participants were given 5 minutes to complete tasks three through six.

### 6.5 Results

**FIGURE 6:**
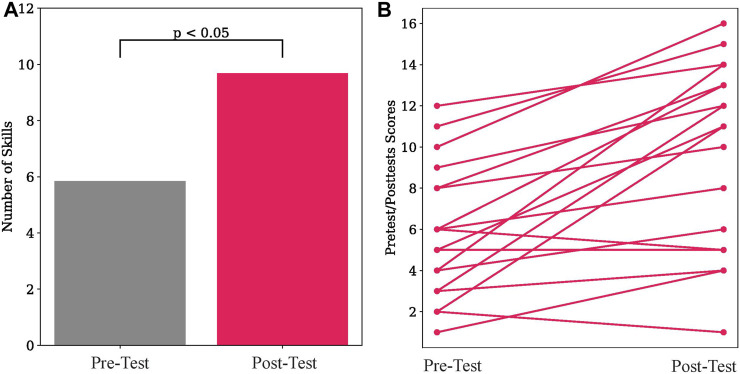
**(A)** Participants demonstrated a significantly higher number of skills in the post-test compared to the pre-test. **(B)** The pre-test and post-test scores for each of the participants.

Participants demonstrated wide variability in their skills on electronic circuits, varying from only demonstrating 6% of skills on the pre-test to showing 71% of skills. Likewise there was a large variation on the post-test with participants varying between 6% and 94%. We compare how many skills the participant correctly demonstrates from the pre-test to the post-test, that is, how much they improved as a result of the robot’s interaction. On average the participant demonstrates correctly 5.83 (*SD* = 3.24) skills on the pre-test, and 9.67 (*SD* = 4.49) skills on the post-test. A *t*-test shows that participants knew significantly more skills during the post-test than during the pre-test (*t* (18) = 8.64, *p* = .006). These results are shown in Figure 6A. Figure 6B shows the improvement of each participant between the pre-test and post-test. 83% of participants improved their skills after the interaction, 6% did not learn any additional skills, and 11% showed fewer skills on the post-test compared to the pre-test. These results show that TD-BKT was able to correctly create a model of the user’s skills and choose appropriate actions to teach each person.

**FIGURE 7:**
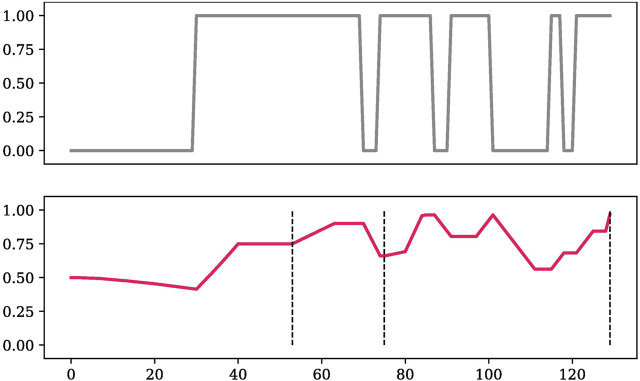
An example of the observation and TD-BKT’s resulting skill estimate of a participant LED’s skill during a task.

In Figure 7, we give an example of how TD-BKT tracked one participant’s LED skill’s estimate. In the observation graph we can see that the person added and removed the LED multiple times during the interaction. This was likely because they were moving the piece around as they were adding new pieces to the circuit. This can also be because the observations were noisy, due to occlusion of the board (The computer vision detected that the user had their hand on top of the board 32% of the time), or due to incorrect observations. The skill estimate graph shows how TD-BKT models the user given the observations. The dashed lines in the figure are the moments the participant pressed the finished button (signalling they believed they had the correct answer or that they were stuck).

We can see that in the figure, the user did not add the LED until around time-step 30, causing many observations with value 0 (skill not demonstrated). Despite these observations, the skill estimate only slowly decreased, and as soon as the user added the LED, the value quickly increased. The user added and removed the LED several times, but nonetheless, TD-BKT kept a high estimate of the user’s skill. At the end of the interaction TD-BKT assumed the user knew the skill, which matched what the user demonstrated.

## 7 Discussion

In this section, we first discuss our proposed solution and its results. In sequence, we present a discussion on the attempted parameter and how it can be used and extended for different use cases. Then, we will discuss several of the limitations of our algorithm. Lastly, we present different scenarios TD-BKT can be applied.

### 7.1 Time-dependant—Bayesian knowledge tracing

In this paper, we have shown that TD-BKT can model a user’s skills during complex tasks. Experiment 1 shows that TD-BKT models a user’s skill more accurately than traditional Bayesian Knowledge Tracing systems. This is because the conventional BKT approach was designed to only model a user’s skills at the end of the task when it has received an unambiguous answer from the user. Whereas TD-BKT considers how long applying each individual skill is expected to take, and therefore can model skills throughout the task. In Experiment 2, it is shown that accurately modeling a user’s skill during the task allows the system to choose good skills to teach a user. Users learn significantly more novel skills with TD-BKT compared to traditional BKT variations. This is essential, as the main goal of tutoring systems is to improve the student’s skills in a particular domain.

Lastly, we validated TD-BKT on a user study with participants building electronic circuit tasks. This demonstrated the applicability of the algorithm to real-world tasks where participant data must be recovered using a sensing system. By modeling users using TD-BKT, the system taught users skills relating to electronic circuit design. Furthermore we have demonstrated that TD-BKT significantly increased participant knowledge on circuits from pre-test to post-test, demonstrating that user’s learned several new skills during the interaction.

### 7.2 Attempted parameter

The attempted parameter captures the expected amount of time before the user would have tried out a skill. This allows TD-BKT to modify the weight of user observations at the start of the interaction and therefore make fewer mistakes. In our algorithm, we assume the attempted parameter is a fixed value throughout the task. However, for future systems, a more advanced computer vision system may be able to provide greater activity resolution by detecting what the user is doing at every time-step. This would provide a more accurate probability that they have attempted each of the skills of the current task. A second addition that we leave as future research is to enhance the attempted parameter by defining order dependencies between the skills. Often the ability of the user to attempt one skill is dependant on another skill being demonstrated first. For example, it is not possible to correctly have an LED on a board in the correct direction before the LED is added. These order dependencies would make the attempted parameter more accurate.

### 7.3 Limitations

Our presented algorithm has several limitations. The first is that we do not consider the order dependencies between different skills, whereas there often is a hierarchical dependency between demonstrating one skill and having knowledge of another. For example, we do not check whether the user has added a music circuit before checking whether they know how to connect the music circuit to a speaker. Future work should investigate how to incorporate hierarchical skill dependencies while the user completes tasks over time.

The second limitation of TD-BKT is that it assumes that each time step is equally important, whereas that might not always be the case during a tutoring scenario. A student might spend some of their time thinking of how they are going to piece their solution together, and then have a burst of action where they demonstrate or attempt all of the skills needed in the task in a short period of time. Future work should analyze how to change the weights of each time step depending on the user’s current actions.

Lastly, we leave as future work applying TD-BKT to a larger variety of domains and learning tasks. Different domains such as learning a new language or learning how to cook, might need the consideration of new parameters to fully represent the data and domain.

### 7.4 Applications

The TD-BKT model is helpful in many different scenarios. The main one (and the one that has been the focus of this paper) is intelligent tutoring systems (ITS). Having an accurate model of a user is essential to tutoring. As ITSs become more predominant and are used for a broader range of tasks and ages, it is vital that not only simple tasks be considered (such as math or multiple choice), but also more intricate task where feedback may be required. Especially in light of the COVID-19 pandemic, ITSs can remove some of the strain on teachers and parents by providing personalized help to a student.

With the spread of collaborative robots in industry, many opportunities arise for these robots to model users and teach while collaborating. Some examples of collaborative tasks include: assembling cars or furniture, building circuits, and doing chemical processing. There are many advantages of creating a model of a human operator in manufacturing. Once the system has an accurate model of each employees skill it can teach additional skills that they might need at work. It can increase workplace safety by taking on dangerous tasks that the user has not yet mastered. And it can do task assignment according to each team members strengths and weaknesses when several people are collaborating.

## Data Availability

The datasets presented in this study can be found in online repositories. The names of the repository/repositories and accession number(s) can be found below: https://github.com/ScazLab/C-BKT.

## Footnotes

1 The code and the data can be found at https://github.com/ScazLab/C-BKT.
